# Molecular Pathology of Pancreatic Cystic Lesions with a Focus on Malignant Progression

**DOI:** 10.3390/cancers16061183

**Published:** 2024-03-18

**Authors:** Yan Hu, Dan Jones, Ashwini K. Esnakula, Somashekar G. Krishna, Wei Chen

**Affiliations:** 1 James Molecular Laboratory, The Ohio State University Comprehensive Cancer Center, Columbus, OH 43210, USA; yan.hu@osumc.edu (Y.H.); daniel.jones@osumc.edu (D.J.); 2 Department of Pathology, The Ohio State University Wexner Medical Center, Columbus, OH 43210, USA; ashwini.esnakula@osumc.edu; 3 Division of Gastroenterology, Hepatology, and Nutrition, The Ohio State University Wexner Medical Center, Columbus, OH 43210, USA; somashekar.krishna@osumc.edu

**Keywords:** pancreatic cancer, molecular mechanisms, IPMN, histologic subtypes, risk stratification, malignant progression, tumorigenesis, EUS nCLE

## Abstract

**Simple Summary:**

Pancreatic cysts are being identified with increasing frequency, and the prevalence increases with age to 10–25% by the 7th decade of life. They also have the potential for malignant transformation. The effective monitoring of the progression of pancreatic cysts and the early detection of pancreatic cancer is crucial. Here, we review recent advances in the molecular pathology of pancreatic cystic lesions and discuss how the revised tumorigenesis and progression model in an intraductal papillary mucinous neoplasm (IPMN) could provide insights into refining strategies for the risk stratification of pancreatic cysts and the early detection of cancer.

**Abstract:**

The malignant progression of pancreatic cystic lesions (PCLs) remains understudied with a knowledge gap, yet its exploration is pivotal for effectively stratifying patient risk and detecting cancer at its earliest stages. Within this review, we delve into the latest discoveries on the molecular level, revealing insights into the IPMN molecular landscape and revised progression model, associated histologic subtypes, and the role of inflammation in the pathogenesis and malignant progression of IPMN. Low-grade PCLs, particularly IPMNs, can develop into high-grade lesions or invasive carcinoma, underscoring the need for long-term surveillance of these lesions if they are not resected. Although *KRAS* and *GNAS* remain the primary oncogenic drivers of neoplastic development in IPMNs, additional genes that are important in tumorigenesis have been recently identified by whole exome sequencing. A more complete understanding of the genes involved in the molecular progression of IPMN is critical for effective monitoring to minimize the risk of malignant progression. Complicating these strategies, IPMNs are also frequently multifocal and multiclonal, as demonstrated by comparative molecular analysis. Algorithms for preoperative cyst sampling and improved radiomic techniques are emerging to model this spatial and temporal genetic heterogeneity better. Here, we review the molecular pathology of PCLs, focusing on changes associated with malignant progression. Developing models of molecular risk stratification in PCLs which can complement radiologic and clinical features, facilitate the early detection of pancreatic cancer, and enable the development of more personalized surveillance and management strategies are summarized.

## 1. Molecular Pathology of Pancreatic Cystic Lesions 

Pancreatic cancer is one of the most lethal cancers and is projected to become the second leading cause of cancer-related deaths by 2030 in the United States [1]. Pancreatic cystic lesions (PCLs) can be generally classified as mucinous neoplastic cysts, non-mucinous neoplastic cysts, and benign non-neoplastic lesions (retention cysts and pseudocysts). Mucinous neoplastic cysts include IPMNs and mucinous cystic neoplasms (MCNs); both are recognized macroscopic precursor lesions of pancreatic cancer. Non-mucinous neoplastic cysts include serous cystadenoma, solid pseudopapillary neoplasm, cystic neuroendocrine tumor, and other rare epithelial cysts (lymphoepithelial cysts, acinar cystic transformation, squamoid cysts, cystic teratomas, etc.). Malignant transformation in these non-mucinous neoplastic cysts is rare but may occur. The molecular alternations of each PCLs are described in detail below, with highlights of abnormalities associated with disease progression (also see Table 1). 

### 1.1. Intraductal Papillary Mucinous Neoplasm (IPMN)

IPMN is a grossly visible (>5 mm) intraductal epithelial neoplasm arising in the main duct and/or branch duct of the pancreas. IPMN can occur anywhere in the pancreatic ductal system, with the pancreatic head being the most common location. Multicentricity (synchronous or metachronous lesions) is observed in up to 40% of cases [2]. The epithelia of IPMN feature papillary formation and abundant mucin production. Histologic subtypes of IPMN are discussed in detail in Section 4.1. 

Somatic activating mutations in *KRAS* and *GNAS* are the most prevalent genetic alterations seen in IPMN, with either or both seen in >95% of all IPMNs [3,4]. A *KRAS* mutation (predominantly codon 12; occasionally codons 13 and 61) is an early event in IPMN tumorigenesis, but the presence of *KRAS* mutations does not correlate with the degree of dysplasia or histologic subtype of IPMNs [5]. Activating *GNAS* mutations affecting codon 201 (aka codon 202) are most prevalent in intestinal-type IPMNs [6,7,8]. In genetically engineered mouse models of pancreatic neoplasia, the presence of *GNAS* mutation alone does not induce the emergence of intestinal features [9,10,11]. Hotspot mutations in *KLF4* (K409 and S411) are recently found in >50% of low-grade IPMNs, but in only 15% of high-grade lesions, suggesting a role in early tumorigenesis [12]. Inactivating mutations of *RNF43*, a negative regulator of the Wnt signaling pathway, are seen in up to 75% of IPMNs [3,13]. *RNF43* mutations are found mainly in non-invasive high-grade lesions and likely represent a later event involved in the progression from low-grade to high-grade lesions in the intestinal pathway [3,7,13,14,15,16,17,18,19]. Both germline and somatic *ATM* mutations have been found in up to 17% of IPMN cases [15]. In addition, alterations in *GLI3*, *SF3B1*, *BRG1*, *LKB1*, *PTEN*, *PI3K*, *PIK3CA*, *STK11*, and genes in the mitogen-activated protein kinase (MAPK) pathway (including *BRAF*, *ERBB2*, *HRAS*, and *MAPK1*) have also been reported in IPMNs [4,15,20,21,22]. Of note, alterations in tumor suppressor genes, particularly *TP53*, p16/*CDKN2A*, *SMAD4*, and *TGFBR2*, as well as mutations of mTOR pathway genes (*PTEN*, *PIK3CA*, and *AKT1*) are associated with advanced neoplasia (high-grade dysplasia and invasion) [23,24,25,26,27,28,29]. *TP53*, *SMAD4*, and *TGFBR2* abnormalities are frequently restricted to invasive carcinoma [15,17]. 

Epigenetic inactivation via DNA methylation is also present in IPMN. The hypermethylation of the *CDKN2A* promoter occurs in >50% of non-invasive IPMNs and IPMNs with associated carcinoma, but methylation is not a common inactivation mechanism for other pancreatic cancer driver genes such as *TP53* and *SMAD4* [30,31]. In both non-invasive IPMNs and IPMNs with associated carcinoma, gene silencing by promoter hypermethylation also occurs in several other genes (*APC*, *CDH1*, *MLH1*, and *MGMT*) [32,33,34,35,36]. Hypermethylation is more prevalent with adenocarcinoma, in which multiple genes could be methylated. Regarding microRNAs, there is a higher expression of miR-21, miR-221, and miR17-3P in IPMNs than in non-mucinous cysts [36].

### 1.2. Mucinous Cystic Neoplasm (MCN)

Similar to IPMNs, a MCN is a mucin-producing cystic epithelial neoplasm, but unlike IPMNs, aMCN is not connected to the pancreatic duct. In addition, a MCN is defined by its characteristic ovarian-type subepithelial stroma. MCNs predominantly (>98%) occur in women and have a predilection for the body/tail of the pancreas. Increasing age correlates with a higher risk of invasive carcinoma, suggesting that progression occurs over a period of years. 

The genetic changes in MCNs are similar to IPMNs, except that *GNAS* mutations are rarely seen in MCNs [23]. Activating *KRAS* mutations are seen in 50–66% of MCNs as well as a loss of function in *RNF43* [8,26,29]. Fewer nonsynonymous somatic mutations are present per MCN, compared to IPMNs and invasive ductal adenocarcinomas [37].

### 1.3. Intraductal Oncocytic Papillary Neoplasm (IOPN)

In the WHO 2019 classification, an IOPN was distinguished from an IPMN due to its distinctive molecular pathogenesis and presenting features. Essentially, all IOPNs have high-grade dysplasia. IOPNs’ complex and arborizing papillae are lined by 2–5 layers of cuboidal to columnar cells with a mitochondrion-rich eosinophilic granular cytoplasm [2]. IOPNs lack most of the previously reported genetic alterations in IPMNs and pancreatic ductal adenocarcinomas, such as mutations in *KRAS*, *GNAS*, *TP53*, and *RNF43* [18,38,39,40,41]. Using a broad RNA-based targeted sequencing panel, Singhi et al. detected rearrangements in *PRKACA* and *PRKACB* in all IOPNs tested, including 20 pancreatic IOPNs and three biliary IOPNs [42]. These include recurrent *ATP1B1-PRKACB*, *DNAJB1-PRKACA*, or *ATP1B1-PRKACA* fusion genes. *ARHGAP26*, *ASXL1*, *EPHA8*, and *ERBB4* have also been reported to be recurrently mutated in some IOPNs [43].

### 1.4. Intraductal Tubulopapillary Neoplasm (ITPN)

Unlike IPMNs, an ITPN is a mucin-poor intraductal neoplasm representing only 3% of all pancreatic intraductal neoplasms. ITPNs are architecturally complex and typically have high-grade dysplasia. They form nodules of back-to-back tubular glands, resulting in large cribriform structures [2]. Most reported alterations related to IPMNs and pancreatic ductal adenocarcinomas, including *KRAS*, *GNAS*, and *BRAF* mutations, are absent in ITPNs [3,44,45,46]. Certain chromatin remodeling genes (*MLL1/2/3*, *BAP1*, *PBRM1*, *EED*, and *ATRX*) and PI3K pathway genes (*PIK3CA*, *PIK3CB*, *INPP4A*, and *PTEN*) are shown to be mutated [44,45,46,47]. A subset of ITPNs harbor *FGFR2* fusion and *STRN-ALK* fusion [47], which are of interest for targeted therapy.

### 1.5. Serous Cystadenoma (SCA)

Unlike the pancreatic intraductal neoplasms, SCA does not connect to the pancreatic ducts and is thought to have a centroacinar origin with the abnormal regulation of the VHL/HIF pathway [2,48]. SCA has a female predominance and a mean size of 4 cm. This benign epithelial neoplasm is composed of uniform cuboidal, glycogen-rich clear cells that often form multilocular, thin-walled cysts containing serous fluid. The microcystic variant shows a characteristic honeycomb or sponge pattern with a central scar in 30% of cases. Rare macrocystic (oligocystic) variants and solid variants also exist. The malignant transformation of SCA is extraordinarily rare, and the diagnosis of malignancy is restricted to cases with unequivocal distant metastasis (almost always to the liver) [2,49].

Germline or somatic alterations of the tumor suppressor gene *VHL* are present in SCAs [18,29,50,51]. The loss of heterozygosity of chromosome 3p at the *VHL* locus also occurs in many sporadic SCAs [36]. In cases with a germline *VHL* mutation, multifocal cysts are common. Alterations in *KRAS*, *GNAS*, *CDKN2A*, and *SMAD4* have not been reported in SCAs [18,36]. *TP53* or *TERT* promoter mutations may be prognostically important as they are associated with the interval growth of the cyst size [52].

### 1.6. Solid Pseudopapillary Neoplasm (SPN)

An SPN is a rare, low-grade malignant tumor that lacks a specific line of pancreatic epithelial differentiation. It occurs predominantly in young women and is theorized to arise from genital ridge cells that were translocated to the pancreas during embryogenesis [53]. SPNs are typically large tumors (average size 8 cm) and may show variable solid and cystic/degenerative components. 

A somatic activating mutation in *CTNNB1* (encoding β-catenin) is the main molecular feature of SPN and occurs in 95% of the cases [18,54,55]. This change leads to the abnormal nuclear accumulation of β-catenin protein. Alterations in *KRAS*, *TP53*, and *SMAD4* have not been reported in SPN [36].

### 1.7. Cystic Neuroendocrine Tumor (Cystic NET)

Well-differentiated NETs are generally slow-growing tumors. They can be classified into a low (G1), intermediate (G2), and high (G3) grade based on the mitotic count and Ki67 proliferative rate [2]. Some NETs can undergo cystic degeneration and present as a PCL. Somatic mutations of *MEN1* and the loss of heterozygosity of *MEN1* occur in 45% and 30–70% of sporadic pancreatic NETs, respectively [36]. The promoter methylation and deletion of *VHL* occur in up to 25% of sporadic pancreatic NETs. Somatic mutations of genes in the mTOR pathway (*PIK3CA*, *PTEN*, and *TSC2*) occur in approximately 15% of sporadic pancreatic NETs [36]. A loss of chromatin remodeling genes *ATRX/DAXX* and the presence of the alternative lengthening of telomeres are associated with a poor prognosis [52]. Pancreatic NETs with a loss of heterozygosity (LOH) of ≥3 genes tend to have distant metastasis [52].

### 1.8. Lymphoepithelial Cysts (LEC) and Other Rare Epithelial Cysts

LECs and other rare benign epithelial cysts, such as acinar cystic transformation (ACT), are in the differential diagnosis of PCLs. These pancreatic cysts can be found accidentally and typically have no risk of malignant transformation. LECs are lined by squamous epithelium and surrounded by subepithelial lymphoid tissue, likely derived from misplaced branchial cleft cysts during embryogenesis [56]. ACTs are non-neoplastic cystic lesion lined by a benign-appearing acinar and ductal epithelium [2]. Chromosomal gains, but not losses, were reported for one ACT case [57], and random X-chromosome inactivation patterns were reported for five other cases [58]. Unlike in neoplastic mucinous cysts and related pancreatic cancers, alterations in *KRAS*, *GNAS*, *RNF43*, *TP53*, *CDKN2A*, and *SMAD4* were not reported in LECs and ACTs [52,59].

## 2. Molecular Pathology of Pancreatic Ductal Adenocarcinoma

Transcriptomics studies based on SNP microarrays and RNA sequencing classified pancreatic ductal adenocarcinomas into two distinct subtypes: basal-like/squamous and classical/progenitor [60,61]. *TP53* mutations are more prevalent in basal-like subtype tumors, whereas *GNAS* mutations are enriched in classical subtype tumors [60]. Unsurprisingly, the basal phenotype is enriched in metastasis and correlated with a worse prognosis [62].

Comprehensive methylation profiling in The Cancer Genome Atlas study identified two clusters of pancreatic ductal adenocarcinomas based on the extent of their DNA hypermethylation and identified almost 100 genes that were recurrently silenced by DNA methylation, including *ZPF82*, *PAPR6*, and *DNAJC*15 [60].

About 10% of patients with pancreatic cancer have a familial basis with germline mutations in certain genes that predispose individuals to pancreatic cancer. This is comprehensively reviewed elsewhere [36,63]. Briefly, well-established pancreatic cancer susceptibility genes include *BRCA2*, *ATM*, *BRCA1*, *PALB2*, *CDKN2A*, *STK11*, *PRSS1*, *SPINK1*, and the mismatch repair genes (*MLH1*, *PMS2*, *MSH2*, and *MSH6*), as summarized in Table 2.

## 3. IPMN Molecular Landscape and Tumorigenesis/Progression Models 

The prevalence of IPMNs ranges from 3 to 6% in the general population, with a higher incidence (>10%) in older adults [64]. Importantly, IPMNs represent detectable and treatable precursor lesions of pancreatic cancer and have been extensively studied as a key model in understanding pancreatic carcinogenesis. Understanding the molecular alterations of precursor lesions will potentially provide helpful guidance in targeting the tumors at an earlier stage and may offer insights into refining strategies for the diagnosis, treatment, and appropriate surveillance of pancreatic lesions [65]. 

### 3.1. Pancreatic Intraepithelial Neoplasia (PanIN), IPMN, and Co-Occurring Invasive Carcinoma

IPMNs are grossly visible PCLs that are larger than 5 mm, according to the 2019 WHO classification. PanINs are microscopic, grossly invisible lesions that are <5 mm. Both PanINs and IPMNs are recognized precursor lesions of pancreatic cancer, and they share morphologic similarities. In the past, IPMNs were defined as ≥1 cm, posing a diagnostic dilemma for the classification of subcentimeter (but >5 mm) mucinous cysts with papillae. It is unclear whether the molecular features for these tiny cysts are more similar to PanINs or IPMNs. Using the acquired *GNAS* hotspot mutation as the prototypic IPMN change, Matthaei et al. studied precursors that were between 2 mm and 7 mm and noted *GNAS* mutations, favoring emerging IPMNs [66]. 

When IPMNs exceed a size of 3 cm, the probability that these cysts harbor malignant changes significantly increases. IPMN-associated invasive carcinoma often presents as a mural nodule. In some cases, an IPMN and an adjacent invasive carcinoma exist side by side, raising the differentials between non-related collision neoplasms or clonal-related lesions. As summarized below, up to 18% of pancreatic ductal adenocarcinomas and co-occurring IPMNs have distinct mutations in cancer driver genes, suggesting that an independent clone may have given rise to invasive carcinoma [17,36,67].

In two independent studies using the exhaustive genetic mapping of human pancreatectomy specimens, Felsenstein et al. [67] and Omori et al. [17] evaluated the clonal relatedness of pancreatic adenocarcinomas and co-occurring IPMNs by comparing their shared mutations [68]. They found that there are three different scenarios in which co-occurring IPMNs and pancreatic cancer are present in the same pancreas: (1) Sequential/related—pancreatic cancer develops directly or sequentially from the IPMNs and they share driver mutations; (2) independent/de novo—pancreatic cancer developed independently from the IPMN and the two lesions are not related and have distinct driver mutations; and (3) the branch-off pathway—invasive cancer and an adjacent IPMN which were initially developed from a common ancestral clone, and then diverged in their subsequent development. 

These different IPMN progression models highlight the importance of the long-term monitoring of IPMNs not only for the malignant progression of IPMNs, but also for independent/de novo cancer in the same pancreas. It should be noted that in the scenario of the de novo pathway, i.e., “neighbors but not relatives”, even if we were to predict the grade of an IPMN with perfect accuracy, we would not be able to rule out an independent cancer arising in the parenchyma adjacent to an IPMN [69]. 

### 3.2. IPMN Molecular Landscape: Genetic Heterogeneity and Multifocal Neoplasia Are Prevalent 

In 1992, Furukawa et al. conducted a detailed morphological study of 3D reconstructed pancreas containing IPMNs, which showed that low-grade neoplastic cells were distributed in a multifocal fashion, while high-grade neoplastic cells were distributed in a unified continuous fashion [70]. These morphological findings match with recent data from the molecular mapping of IPMNs. Several recent independent sequencing studies using the laser capture microdissection of multiple regions of IPMNs and single cells revealed prevalent genetic heterogeneity in driver gene mutations in early lesions yet less heterogeneity in late-stage lesions [15,71,72]. 

**Figure 1:**
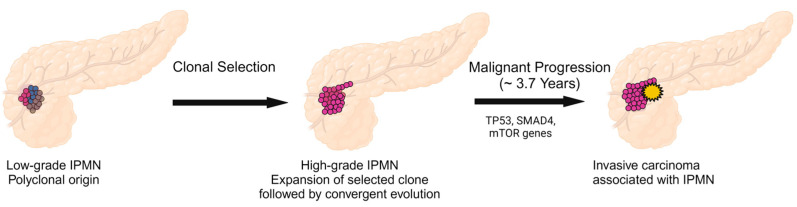
Revised Progression Model of Intraductal Papillary Mucinous Neoplasm.

In a cohort of 227 neoplastic tissue samples from 20 surgically resected IPMNs, Fischer et al. investigated the genetic evolution of IPMNs by the multiregional sequencing of early- and late-stage lesions [71]. IPMNs with low-grade dysplasia have significantly more heterogeneity with respect to the early drivers *KRAS* and *GNAS* compared with IPMNs with high-grade dysplasia. For example, low-grade IPMNs often have multiple mutations in *KRAS*, ranging from 2 to 4 per IPMN, representing either a multifocal or branched origin of IPMN tumorigenesis. In contrast, high-grade IPMNs lack this heterogeneity and there is evidence for the convergent evolution of mutations in *RNF43* and *TP53*, which are acquired during later stages of tumorigenesis. In addition, RNA in situ hybridization confirms the spatial separation of mutant *KRAS* subclones within the IPMN. These sequencing data, coupled with evolutionary modeling, have led some to revise IPMN tumorigenesis. In this model, IPMNs originate from multiple clones that evolve independently, one of which may acquire additional driver gene mutations that lead to clonal expansion and progression to advanced neoplasia [71] (Figure 1). This supports the presence of some type of field effect affecting the pancreatic ductal system, such as the germline genetic background or external risk factors (e.g., inflammation), that leads to this multifocality in these pancreatic precancerous lesions [73]. 

In another large study of 148 neoplastic lesions (IPMNs, MCNs, and small associated invasive carcinomas) from 18 patients using whole exome or targeted sequencing, Noe et al. provided molecular proof that non-invasive IPMNs and MCNs are precursors of invasive pancreatic cancer [15]. This was evidenced by multiple shared driver and passenger mutations between the non-invasive and invasive components. In addition, evolutionary analyses by Bayesian hierarchical models suggested an average window of 3.7 years between the development of high-grade dysplasia and invasive pancreatic cancer (Figure 1) [15], highlighting an opportunity for detection and treatment before malignant transformation. 

## 4. Histologic Subtypes of IPMN and Tumorigenesis

### 4.1. Histologic Subtypes of IPMN

IPMNs are currently classified into three different epithelial subtypes: gastric, intestinal, and pancreaticobiliary. The latter two subtypes carry a higher risk of malignant transformation [74]. The pattern of expression of apomucins MUC1, MUC2, MUC5AC, and MUC6 can be used to help classify IPMN epithelial subtypes. The MUC expression profile and characteristics of the different pancreatic intraductal neoplasms are summarized in Table 3.

### 4.2. Relationship of Different Epithelial Subtypes of IPMN and Malignant Progression

It has long been observed that IPMNs, not uncommonly, have a mixed histology, and the gastric-type epithelium is often associated with other epithelial subtypes [5,75,76]. In an elegant study by Omori et al., 48 (60%) of 60 intestinal-type IPMNs showed a morphological transition from the gastric-type epithelium to the intestinal-type epithelium, and the histologic observations are further supported by immunohistochemical and molecular findings [16]. Caudal-type homeobox 2 (CDX2) is a transcription factor expressed in the intestinal epithelium and may function as a coordinator of intestinal differentiation in IPMNs [16]. It was demonstrated that the expression of CDX2 in a morphologically gastric-type epithelium predates the transition to an intestinal phenotype. In addition, the intestinal-type epithelium harbored frequent *GNAS* and *RNF43* mutations and shared identical *GNAS* and *KRAS* mutations with the concurrent gastric-type epithelium. These findings lead to the hypothesis that the gastric-type epitheliums that acquire *GNAS* mutations together with the induction of CDX2 expression may evolve with clonal selection and additional molecular alterations such as *RNF43* into intestinal-type IPMNs [16]. Furthermore, it has long been suggested that the pancreaticobiliary type is a variant of the high-grade gastric type [76]. 

All three epithelial subtypes share the expression of the MUC5AC protein, a defining MUC protein for the gastric subtype. This universal expression of the MUC5AC protein is another clue that gastric-type lesions are a common ancestral lesion of IPMNs that may develop into other non-gastric epithelial subtypes. MUC5AC levels in the serum are also significantly increased in high-risk IPMN patients [77]. In a cohort of 107 resected IPMNs, 83% exhibited gastric-type lesions, and all IPMNs with multiple epithelial types contained a gastric-type epithelium [78]. By comparing shared driver gene mutations, the authors suggested that low-grade gastric-type lesions harboring both *KRAS* and *GNAS* mutations are likely to progress into intestinal-type lesions, while those harboring only *KRAS* mutations are likely to progress to pancreaticobiliary lesions [78].

The hypothesis that gastric-type lesions likely represent the ancestral lesion of intestinal-type IPMNs aligns logically with the perspective of pathophysiology. Embryonically, both the stomach and pancreas are derived from the foregut. In the carcinogenesis of the stomach, stepwise changes from the normal gastric foveolar epithelium to the metaplastic intestinal epithelium, dysplasia, and carcinoma are often observed. It is reasonable that the pancreatic ductal epithelium may adopt a similar carcinogenesis pathway. Being anatomically downstream to the stomach and pancreas, the mid-gut-derived intestinal epithelium is biologically designed to tolerate the harsh digestive juice (gastric acid and pancreaticobiliary secretions). Therefore, the injured gastric and pancreatic epithelia may resort to a more “protective” metaplastic intestinal epithelium. If the insults are not removed and the epithelial damage is ongoing, dysplasia and cancer may eventually develop. However, some gastric-type IPMNs may directly progress to high-grade pancreaticobiliary IPMNs without intestinal metaplasia. 

Mas et al. proposed two hypothetical IPMN progression pathways: intestinal and non-intestinal (gastric/pancreaticobiliary) pathways. In the intestinal pathway, *KRAS* and *GNAS* alterations drive the initiation, and then the IPMN progresses under the influence of *CDX2* and *RNF43* mutations via the *GNAS*-driven pathway to invasive colloid carcinoma. In the non-intestinal pathway, *KRAS* with *PTEN* or *LKB1* mutations lead to *KRAS*-driven IPMN progression with additional mutations (*SMAD4*, *TP53*, or *CDKN2A*) to tubular carcinoma [74]. 

Due to the risk of low-grade gastric IPMNs advancing to other high-grade subtypes, the ongoing surveillance of low-grade gastric IPMNs is necessary. On the other hand, if epithelial subtyping can be performed preoperatively, for example, using EUS-nCLE endomicroscopy, the distinction of the gastric-type epithelium from other high-grade epithelial subtypes will serve as a promising preoperative risk stratification tool.

## 5. The Role of Inflammation in the Pathogenesis and Malignant Progression of IPMN

The role of inflammation in pancreatic carcinogenesis has long been observed. Longstanding chronic pancreatitis is a well-known risk factor for pancreatic cancer [2]. Repeated episodes of inflammation, injury, and metaplasia/repair are thought to drive neoplasia [79]. This is exemplified in individuals with cystic fibrosis and patients with hereditary pancreatitis due to mutations in *PRSS1* or *SPINK1*, who have an increased risk of pancreatic cancer due to chronic inflammation and related changes in the pancreas [2,79,80]. In a cohort of 150 patients with resected IPMNs, Tsutsumi et al. showed that the patients with acute pancreatitis (AP) had a significantly higher frequency of carcinoma derived from IPMNs than those without AP [81]. Xu’s study of 47 IPMN patients revealed a higher risk of high-grade dysplasia in those with AP compared to those without [82]. Takenake et al. prospectively examined 69 consecutive patients with IPMNs and detected a higher rate of invasive IPMN in patients with an EUS finding of chronic pancreatitis (CP) in the background pancreatic parenchyma [83]. Unsurprisingly, promoters of chronic inflammation, such as smoking and obesity, have been identified as factors associated with IPMN progression [84]. In addition, studies on cyst fluid inflammatory markers showed a positive correlation with the dysplasia grade of IPMNs. Higher levels of markers like PGE2 and Interleukin (IL)-1β were found in patients with high-grade or invasive IPMNs compared to those with low-grade IPMNs [85,86]. Sadot et al. observed a positive correlation between neutrophil infiltration and the dysplasia grade in IPMN [87].

While there is a strong association between inflammation and advanced neoplasia in IPMNs, the relationship between pancreatitis and the initiation of IPMN pathogenesis remains less clear. It is uncertain whether AP or CP serve as the cause of IPMNs or are instead a consequence of its presence. In a retrospective study of 489 patients with IPMN, Jang et al. concluded that acute (recurrent) pancreatitis caused by IPMNs was infrequent (7%) and more prevalent in the main-duct or combined-duct type IPMN [88]. In a cohort of 390 patients with IPMNs and 390 matched controls, Capurso et al. found that patients with a history of CP had a higher incidence of IPMNs compared to the controls (3.1% vs. 0.3%), suggesting that CP might be an independent risk factor for developing IPMNs [89]. However, Talamini et al. conducted a prospective study following 473 patients with CP and found that in general, patients with IPMNs present different epidemiological characteristics than those with CP. They suggested that in most cases, IPMN is the cause of CP and not vice versa [90]. Further research is needed to clarify the complex interplay between pancreatitis and IPMN pathogenesis. 

Another controversial issue is whether the presence of the microbiome within the IPMN is the driver of the inflammatory response/dysplasia progression in the cyst. Some prior studies suggested that microbiota may be a contributing factor to the progression of IPMNs. Gaiser et al. demonstrated that *Fusobacterium nucleatum* (an oral bacterium), was detected at a significantly higher rate in patients with high-grade IPMNs than those with non-IPMN pancreatic cysts [91]. Hozaka et al. found that increased proportions of Proteobacteria and Fusobacteria correlated with an increasing percentage of intestinal subtype IPMNs. Fusobacteria were abundant in the intestinal type of invasive main duct IPMNs. These findings suggested that the intratumoral microbiota may be involved in tissue differentiation and the progression of IPMNs [92]. However, most recent data using cyst fluid obtained intraoperatively indicated that there is no microbiome within IPMNs, and the presence of bacteria within outliner cyst fluid samples is mainly related to prior invasive intervention [93].

## 6. Advanced Diagnostic Tools for Pancreatic Cystic Lesions

Despite our increasing knowledge of PCLs, the continued resection of benign cysts and the resection of IPMNs with low-grade dysplasia remain. Ongoing research and efforts are focused on developing advanced diagnostic tools and techniques for more accurate risk stratification.

### 6.1. Advanced Analysis of Cyst Fluid/Biopsy/Serum 

Mutations and metabolic changes in neoplastic cells can be detected in aspirated cyst fluid, pancreatic biopsy, or serum; therefore, analyses of such samples are a promising diagnostic tool to classify and risk-stratify PCLs preoperatively. The array of possible analytes is expanding and includes neoplastic nucleic acids (driver gene mutations by next-generation sequencing [NGS], methylation profiles, and microRNA), proteins, and metabolites.

According to the Kyoto guidelines for the management of IPMNs [94], sensitivities and specificities to diagnose mucinous cysts using cyst fluid biomarkers are 58% and 87% by CEA (cutoff value > 192 ng/mL), and 93% and 89% by glucose (cutoff value < 50 ng/dL), respectively. However, neither cyst fluid CEA nor glucose levels are helpful in identifying cysts with high-grade dysplasia or invasive carcinoma [95]. 

NGS testing of pancreatic cyst fluid has been clinically proven to be a useful diagnostic tool, although it is not widely available. Given that over 95% of IPMNs exhibit somatic mutation(s) in *KRAS* and/or *GNAS*, the molecular analyses of cyst fluid for these mutations serve as a highly sensitive assay for the identification of IPMNs [4,36,52,72]. A comprehensive systemic review and meta-analysis showed that mutations in *KRAS* and/or *GNAS* allowed the identification of mucinous cysts with a sensitivity of 79% and specificity of 98%, using pooled data from 1185 surgical resections of PCLs [96]. Genetic alterations in *CDKN2A*, *PIK3CA*, *SMAD4*, and *TP53* can serve as biomarkers for detecting advanced neoplasia, with sensitivities of 11%, 10%, 9%, and 42%, and specificities of 97%, 97%, 98%, and 95%, respectively, based on 666 surgically resected PCLs [50,59,96]. The combination of MAPK/GNAS and TP53/SMAD4/CTNNB1/mTOR alterations demonstrated 88% sensitivity and 98% specificity for advanced neoplasia in PCLs [52]. Furthermore, new technology allows highly sensitive assays to detect low-frequency mutations in a minimal amount of samples. A multiplex digital PCR assay has been reported to simultaneously identify multiple *KRAS* and *GNAS* mutations in small residual specimen such as fine-needle aspiration needle flush [97]. The DNA-based testing of serum/liquid biopsy is emerging. Hotspot *GNAS* mutation was detected in circulating cell-free DNA from blood samples of IPMN patients but not in patients with SCAs or patients without pancreatic lesions, suggesting the potential utility of cell-free DNA in IPMN diagnosis and disease monitoring [98]. 

As discussed above, DNA methylation patterns have been described in IPMNs [35,99,100,101,102,103], and methylation profiles are detectable in cyst fluid [100]. The methylation of certain genes, including *BNIP3*, *CDO1*, *EBF3*, *NXPH1*, *PTCHD2*, and *SOX17*, have been reported to be more prevalent in high-grade IPMNs than in low-grade ones [101,103]. Methylation profiling is also emerging for pancreatic neuroendocrine neoplasms [65]. 

The expression of microRNAs has been investigated as a potential diagnostic approach in pancreatic cyst fluid as a marker for the malignant transformation of IPMNs. A panel of six miRNAs (miR-711, miR-3679-5p, miR-6126, miR-6780b-5p, miR-6798-5p, and miR-6879-5p) were found to be significantly enriched in IPMN-associated adenocarcinoma compared to those in non-invasive IPMNs [104]. The utility of microRNAs in different types of body fluids (urine, serum, or saliva) has also been studied as biomarkers for the early detection of pancreatic cancer [105].

Other small molecules in the pancreatic cystic fluid can also be used as biomarkers for cyst classification and risk stratification. For instance, a recent metabolic profiling study of pancreatic cyst fluid by untargeted mass spectrometry and quantitative nuclear magnetic resonance showed that (Iso)-butyrylcarnitine alone has a diagnostic accuracy of 89% to separate malignant from benign pancreatic cysts, and 5-oxoproline alone has a diagnostic accuracy of 90% to differentiate mucinous from non-mucinous cysts [106]. 

### 6.2. Endoscopic Ultrasound and Ancillary Techniques 

Endoscopic ultrasound (EUS) not only functions as a means to obtain pancreatic cystic fluid for the aforementioned analysis but also stands out as a more sensitive technique (compared to CT or MRI) for assessing PCLs [107]. EUS has added advantages when contrast agents are used or coupled with other ancillary techniques. 

#### 6.2.1. Contrast Enhanced-Endoscopic Ultrasound (CE-EUS)

CE-EUS can provide information on tissue microvascularization for differentiating enhanced from non-enhanced (mucus plugs or cellular debris) mural nodules. Mural nodules carry a 9.3-fold increased risk of malignancy if present in an IPMN [108], and its characterization is important. In a meta-analysis involving 532 patients aimed at evaluating the diagnostic performance of CE-EUS in identifying advanced neoplasia within mural nodules, CE-EUS demonstrated a pooled sensitivity of 88.2%, a specificity of 79.1%, and diagnostic accuracy of 89.6% [109]. However, there remains interobserver variability in interpreting CE-EUS results.

#### 6.2.2. Endoscopic Ultrasound Guided Needle Confocal Laser Endomicroscopy (EUS-nCLE)

When EUS is coupled with needle-based confocal laser endomicroscopy (nCLE), it can provide real-time imaging of the internal epithelium of pancreatic cysts and provide an excellent diagnosis of PCLs with an accuracy of over 90% [110,111,112]. In addition, nCLE evaluation of the “thickness” and “darkness” of the cyst epithelia allows the grading of dysplasia and thus risk stratification of IPMNs [113], which can be further augmented by machine learning/artificial intelligence (AI) [114,115,116]. In the analysis of high-yield (edited) EUS-nCLE videos using a preliminary AI model, a higher accuracy rate of 82% was achieved for detecting advanced neoplasia compared to the AGA guidelines (68.6%) and Fukuoka criteria (74.3%) [116]. Our current research is centered around the highly accurate risk-stratification of IPMNs through the utilization of EUS-nCLE. We aim to create a comprehensive diagnostic tool for evaluating PCLs by combining EUS techniques with cyst fluid NGS analysis, morphometric analysis from nCLE, and the integration of AI. This combined approach has the potential to significantly enhance the accuracy and effectiveness of PCL evaluation.

In a prospective study involving 65 subjects with reference histopathology, EUS-nCLE was compared to cyst fluid CEA and cytology analysis to differentiate mucinous from non-mucinous PCLs. The study found that EUS-nCLE demonstrated higher accuracy, with 98% sensitivity, 94% specificity, and 97% accuracy, compared to CEA and cytology analysis. This indicates that EUS with nCLE can effectively differentiate between mucinous and non-mucinous PCLs [112]. 

Another study involved 76 EUS-nCLE videos of PCLs, and a panel of 13 experienced endosonographers demonstrated “almost perfect” interobserver agreement (IOA, κ = 0.82, 95% CI 0.77–0.87) and intra-observer reliability (IOR, κ = 0.82, 95% CI 0.78–0.85) for distinguishing mucinous from non-mucinous PCLs. The highest IOA was observed for SCAs, followed by IPMNs. The accuracy of EUS-nCLE in diagnosing PCL subtypes was high for non-mucinous cysts (SCA: 98%; cystic-NET/SPN: 96%; pseudocyst: 96%), and slightly lower for mucinous lesions (IPMN: 86%; MCN: 84%) [117].

In an EUS-nCLE morphometric study, researchers aimed to establish EUS-guided nCLE criteria for differentiating IPMNs with high-grade dysplasia/adenocarcinoma (HGD-Ca) from those with low/intermediate-grade dysplasia (LGD). Through a multi-phase analysis, they identified an increased papillary epithelial width and darkness as the most sensitive and accurate variables for detecting HGD-Ca. Logistic regression models showed high sensitivity and specificity for these variables, providing quantifiable criteria that can aid in the risk stratification of IPMNs [113].

In summary, EUS-nCLE has shown promising results in differentiating mucinous from non-mucinous PCLs, with high accuracy and interobserver agreement. Additionally, the technique holds the potential for improving the diagnosis and risk stratification of IPMNs, as demonstrated by the development of computer-aided diagnosis (CAD) and AI algorithms. Further validation studies and enhancements to the AI models are necessary to fully exploit the benefits of EUS-nCLE in clinical practice.

#### 6.2.3. Endoscopic Ultrasound-Guided Through-The-Needle Biopsy (EUS-TTNB)

EUS-TTNB permits “bite” biopsies of the cyst wall by a microforceps introduced through a standard EUS 19-gauge FNA needle. TTNB generates larger tissue fragments and has higher diagnostic yields (56.2% to 69.5%) compared to EUS-FNA (20.8% to 29.5%), according to a prospective study and two meta-analyses [118,119,120]. In three meta-analyses of EUS-TTNB (a total of 27 studies, 979 patients), the pooled sensitivity and specificity for the diagnosis of PCLs/mucinous cysts are 86–90.1% and 94–95%, respectively [119,120,121]. However, a relatively high rate of complications such as acute pancreatitis and intra-cystic hemorrhage (7 to 9.9%) have been reported with mucinous cysts or cysts with a connection to the pancreatic duct [118,119,120]. In a multicenter European study, the risk of acute pancreatitis in IPMNs was estimated to be 28% of those undergoing EUS-TTNB and sampled with multiple microforceps passes [122]. Therefore, at our institution, we exclusively reserve TTNB for patients where the necessity of an accurate diagnosis outweighs the risks, typically in cases when nCLE shows SCAs/non-mucinous patterns. 

## 7. Conclusions and Future Directions

There have been great advancements in understanding the molecular underpinning of tumorigenesis and the malignant progression of PCLs. The genetic heterogeneity of IPMNs presents a substantial challenge to preoperative management, necessitating adjustments in our approaches to preoperative sampling for the improved coverage of spatial and temporal genetic heterogeneity. Multifocal biopsies of the cyst wall and serial NGS testing of cyst fluid may be of value in surveillance and risk stratification. Newer and emerging advanced cyst fluid/biopsy/serum tests to detect neoplastic driver mutations, methylation profiles, microRNA, and changes in proteins and metabolites hold great promise in improving the diagnosis and management of PCLs. The evidence suggests that low-grade gastric-subtype IPMNs may develop into other high-grade subtypes (intestinal and pancreaticobiliary), underscoring the need for the long-term surveillance of the low-grade gastric-type IPMNs. While cyst fluid analysis represents a more generous and inclusive sample than a single bite forceps biopsy, it is still imperfect due to the genetic heterogeneity in IPMNs. One way to solve this problem is to develop AI-augmented imaging techniques to examine the entire cyst epithelium in real-time. In the future, an ensembled approach using clinical features, radiomics, cytopathology, advanced cyst fluid analysis (genetic and biochemical biomarkers), and EUS-nCLE may provide the most accurate evaluation of PCLs. Future research is needed to identify the driving forces behind the field phenomenon of tumorigenesis and malignant progression in IPMNs. 

## Figures and Tables

**Table 1:** Summary of Key Molecular Alternations in Pancreatic Cystic Lesions.

Pancreatic Cystic Lesions	Molecular Alterations	
	Most Common	Enriched in Advanced Neoplasia */Progression
**Mucinous Neoplastic Cysts**		
Intraductal papillary mucinous neoplasm (IPMN)	*KRAS*, *GNAS*, *KLF4*, *RNF43*, (rare *BRAF*, *EGFR*, *HER2)*	*TP53*, *SMAD4*, mTOR genes **
Mucinous cystic neoplasm (MCN)	*KRAS*, *RNF43*	*TP53*, *SMAD4*, mTOR genes **
Intraductal oncocytic papillary neoplasm (IOPN)	Rearrangements in *PRKACA* and *PRKACB*; Mutations in *ARHGAP26*, *ASXL1*, *EPHA8*, *ERBB4*	
Intraductal tubulopapillary neoplasm (ITPN) ***	Chromatin remodeling genes (*MLL1*, *MLL2*, *MLL3*, *BAP1*); PI3K pathway genes (*PIK3CA*, *PTEN*)	
**Non-Mucinous Neoplastic Cysts**		
Serous cystadenoma	*VHL*	*TP53*, *TERT* promoter mutations
Solid pseudopapillary neoplasm	*CTNNB1*	
Cystic neuroendocrine tumor	*MEN1* *VHL*	Loss of *ATRX*/*DAXX*; Alternative lengthening of telomeres; Loss of heterozygosity ≥ 3 genes

* Advanced neoplasia = High-grade dysplasia and invasive carcinoma; ** mTOR genes include *PTEN*, *PIK3CA*, *AKT1*: *** ITPN is a mucin-poor neoplasm.

**Table 2:** Hereditary pancreatic cancer genes and risk of pancreatic cancer.

Affected Gene(s)	Hereditary Syndrome	Estimated Lifetime Risk of Pancreatic Ductal Adenocarcinoma
*STK11*	Peutz–Jeghers syndrome	30–60%
*PRSS1*	Hereditary pancreatitis	≥30–40%
*SPINK1*, *CPA1*, or *CPB1*	Hereditary pancreatitis	Lower than for *PRSS1*
*CDKN2A*	Familial atypical multiple mole melanoma	15–25%
*BRCA2* and *BRCA1*	Hereditary breast and ovarian cancer syndrome	3–10% & 2–5% respectively
*PALB2*	Hereditary breast and ovarian cancer syndrome	7.5%
Mismatch repair genes	Lynch syndrome	6% (*MSH2*)
*ATM*	None	4%

Adapted from ref. [2].

**Table 3:** Characteristics of Pancreatic Intraductal Neoplasms.

	IPMN			IOPN	ITPN
	Gastric	Intestinal	Pancreaticobiliary		
Prevalence	50–60% of IPMN	20–30% of IPMN	10–15% of IPMN	4.5% of all pancreatic intraductal neoplasms	3% of all pancreatic intraductal neoplasms
Commonly involved duct	Branch	Main	Main	Main and/or Branch	Main
Dysplasia grade	Low	High	High	High	High
MUC IHC	MUC1− MUC2− MUC5AC+ MUC6−/+	MUC1− MUC2+ MUC5AC+ MUC6−	MUC1+ MUC2− MUC5AC+ MUC6+	MUC1+ MUC2− * MUC5AC+ MUC6+	MUC1+ MUC2− MUC5AC− MUC6+
Associated carcinoma	Tubular carcinoma	Colloid carcinoma	Tubular carcinoma	May resemble colloid carcinoma	Tubular carcinoma
Molecular Features	*KRAS*-driven pathway	*GNAS*-driven pathway	*KRAS*-driven pathway	Rearrangements in *PRKACA* and *PRKACB*	Chromatin remodeling genes, PI3K pathways genes, Gene fusions

* Focal MUC2 expression may be present in goblet cells. IPMN = Intraductal Papillary Mucinous Neoplasm, IOPN = Intraductal Oxyntic Papillary Neoplasm, ITPN = Intraductal TubuloPapillary Neoplasm.
